# Efficacy of Transcranial Direct Current Stimulation in Developmental Dyslexia: a scoping review

**DOI:** 10.1590/2317-1782/e20240134en

**Published:** 2025-02-24

**Authors:** Esther Constantino, Isabela Ciola de Castro, Vânia Lúcia Carvalho de Lima, Clara Brandão de Avila

**Affiliations:** 1 Departamento de Fonoaudiologia, Escola Paulista de Medicina – EPM, Universidade Federal de São Paulo – UNIFESP - São Paulo (SP), Brasil.; 2 Programa de Pós-graduação em Distúrbios da Comunicação Humana, Universidade Federal de São Paulo – UNIFESP - São Paulo (SP), Brasil.

**Keywords:** Transcranial Direct Current Stimulation, Developmental Dyslexia, Reading, Rehabilitation, Systematic Review

## Abstract

**Purpose:**

This study aims to improve the existing knowledge about the application of Transcranial Direct Current Stimulation in rehabilitating Developmental Dyslexia, both alone and in conjunction with other therapeutic approaches.

**Research strategies:**

The research was carried on the PubMed, Elsevier, LILACS and ERIC – Institute of Education Science.

**Selection criteria:**

Peer-reviewed journal articles were included if published in English, Portuguese, and Spanish and be broken down from the research question devised by the PICO acronym.

**Data analysis:**

Specific data collected according to delineation, summarized by descriptive analysis.

**Results:**

Eleven articles were analyzed. Five of them associated tDCS with cognitive-linguistic or reading stimulation therapy. Assembly and application frequency parameters varied. The results indicated a positive effect on reading skills after the intervention in all of them.

**Conclusion:**

The selected studies showed an improvement in reading speed and accuracy after active transcranial direct current stimulation, whether or not it combined with other cognitive-linguistic and reading stimulation. In some cases, the positive effects persisted up to six months after the intervention, making this a tool that can be used in the treatment of individuals with dyslexia.

## INTRODUCTION

Developmental Dyslexia (DD) is a neurodevelopmental disorder characterized by reading difficulties, mainly related to decoding, with possible consequences for reading comprehension^(1)^. A multidisciplinary team performs its diagnosis and must mainly identify manifestations of deficits in processing phonological information^(2)^. Searching for effective intervention strategies is vital given the high DD prevalence and its significant impact on education and personal and professional development.

An overview of intervention approaches for developmental dyslexia (DD) reveals that they primarily focus on the stimulation and training of cognitive skills, including phonological and reading interventions. These interventions often incorporate multisensory stimulation methods, such as auditory-visual-motor integration^(3)^, vestibular activities^(4)^, perceptual auditory training^(5)^, and targeted auditory stimulation^(6)^.

However, a systematic review study on DD pointed out the difficulty in achieving substantial positive effects in stimulation for reading rehabilitation^(7)^. The effect size found is almost always small due to the persistent condition typical of DD, with invariably slow clinical evolution^(1)^ and the difficulty of carrying out clinical trials with large samples^(7)^.

As a result, direct current transcranial stimulation (tDCS) emerged as a promising technique for the treatment of DD. This is an innovative, safe and noninvasive method, which through two electrodes of different polarities and conductive sponges placed on the scalp, employs a low -intensity electric current (0.8ma to 2ma) and according to its placement (anodic electrode location, responsible for awakening the area and reducing the threshold of the action and cathode potential, responsible for inhibiting the area, ie increasing the threshold) can modulate neuronal activity in specific brain areas^(8-10)^, and, given the above, the possibility of its use in patients with DD is questioned^(11-13)^.

The use of tDCS in clinical practice brings new possibilities for intervention in DD, especially for speech therapists who are specialized and trained in its application. It is vital to evaluate different types of therapy and their respective effects on reading skills given the lack of consensus on combining reading stimulation with tDCS. Assessing application parameters, including the number of sessions needed, is also crucial to ensure a lasting effect.

This study aims to improve and consolidate the existing knowledge about the application of Transcranial Direct Current Stimulation (tDCS) in rehabilitating Developmental Dyslexia (DD), considering its application in isolation and combination with other therapeutic approaches. The primary objective is to clarify doubts and guide speech therapists and other professionals involved in DD rehabilitation on this therapeutic approach’s effectiveness and correct application. To achieve this, information was compiled on the procedures and effectiveness of tDCS combined with different therapeutic stimulation protocols for DD. To achieve this, information was compiled on the procedures and effectiveness of tDCS combined with different therapeutic stimulation protocols for DD.

This scoping review was based on the hypothesis that knowing the tDCS application parameters in the rehabilitation of DD will provide some necessary knowledge about the effect of its application and contribute to elaborating effective protocols. This scoping review’s results will also produce a solid basis for developing objective intervention strategies. Thus, this work seeks to offer significant inputs to the field of DD rehabilitation, promoting advances in scientific knowledge and clinical practice.

## METHODS

This scoping review aimed to map and explore research on this subject in the literature, following the recommendations of the Preferred Reporting Items for Systematic Reviews and Meta-Analyses extension for Scoping Reviews (PRISMA-ScR)^(14)^ and the framework to accomplish this effectively^(15-17)^. This framework consists of five steps: 1) identification of the research question; 2) identification of relevant studies; 3) selection of studies for review; 4) data mapping; and 5) collection, summary, and reporting of results. The steps were performed as described below:

### Identification of the research question

The research question was formulated based on the PICO (Population, Intervention, Comparison, and Outcome) structure, which helps focus the question and determine the relevant aspects of the topic that will be examined. In this case, our PICO question was: In patients with Developmental Dyslexia (P), therapies such as Transcranial Direct Current Stimulation (tDCS) (I), compared qualitatively regarding arrangements, protocols and effects (C), affects the effectiveness of reading rehabilitation (O)?”

### Identification of relevant studies

A high-sensitivity search was performed in the following databases to identify potentially relevant papers: PubMed via NLM – 1954 to 2023, Embase via Elsevier – 1966 to 2023, LILACS – via BVS, from 1980 to 2023, and ERIC – Institute of Education Science – 1994 to 2023. The elaboration of the search strategy followed the recommendation of the Peer Review of Electronic Search – PRESS^(18)^, assisted by an experienced professional. Initially, words found in the titles and abstracts of relevant articles and the indexing terms adopted to describe them were used to develop a comprehensive search strategy. The search strategy, including all identified keywords and indexing terms, was adapted for each database. The search terms were “transcranial direct current stimulation” [Title/Abstract], AND dyslexia [Title/Abstract], and “transcranial direct current”. Moreover, the reference list of all included sources of evidence was examined for additional studies.

Different types of studies were analyzed: experimental and quasi-experimental studies, including randomized clinical trials, non-randomized clinical trials, before-and-after studies, and interrupted time series studies, analytical observational studies, including prospective and retrospective cohort studies, case-control studies, and analytical cross-sectional studies, descriptive observational studies, such as case series, individual case reports, descriptive cross-sectional studies, and systematic reviews that meet the inclusion criteria, depending on the research question.

### Selection of studies for review

Peer-reviewed journal articles were included if published in English, Portuguese, and Spanish.

The eligible criteria can be broken down as follows from the research question devised by the PICO acronym:

1. **Population (P)**: Patients diagnosed with Developmental Dyslexia. This group may include children, adolescents, and adults with a confirmed diagnosis of DD;
2. **Intervention (I)**: Studies using Transcranial Direct Current Stimulation (tDCS) as an intervention method. The established criterion is that the intervention must be carried out through tDCS, regardless of the specific application protocol or the duration of treatment;
3. **Comparison (C):** studies that compared active and sham groups, different cerebral areas stimulated, and longitudinal studies that compared outcomes at different times;
4. **Outcome (O)**: The main inclusion criterion is reading rehabilitation effectiveness. Eligible studies must have evaluated and reported the effects of tDCS (or the comparative intervention) on reading or reading-related skills. They include measures of reading fluency, reading comprehension, reading speed, decoding accuracy, phonological processing skills, and other related outcomes.

### Data mapping

**Figure 1:**
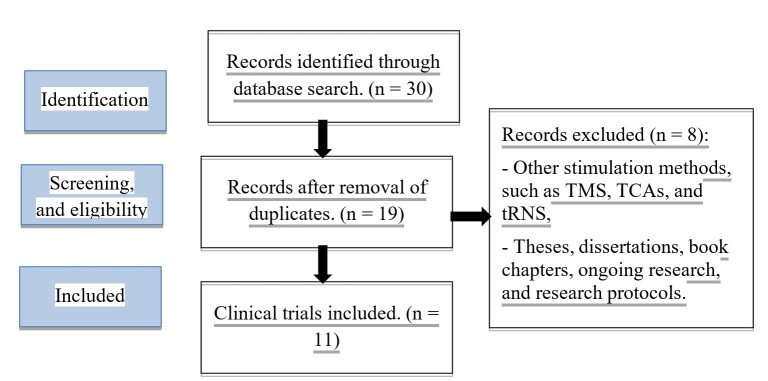
Flowchart: Literature search and screening process (PRISMA)

After the search, all identified citations were collected and entered into Rayyan Systematic Review, removing duplicates. After a pilot test, two or more independent reviewers analyzed the titles and abstracts according to the inclusion criteria established for the review. Two or more independent reviewers assessed the full text of selected citations in detail against the inclusion criteria. The reasons for excluding sources of evidence that do not meet the inclusion criteria were recorded and reported in the scoping review. Any disagreement between reviewers at each stage of the selection process was resolved through discussion or with the participation of an additional reviewer. The results of the search and the inclusion process of the studies were fully reported in the final scoping review and presented in a flowchart (Figure 1) of the PRISMA-ScR (Preferred Reporting Items for Systematic Reviews and Meta-Analyses Extension for Scoping Review)^(14)^.

**Caption:** TMS: Transcranial Magnetic Stimulation; tACS:Transcranial Alternating Current Stimulation; tRNS: Transcranial Random Noise Stimulation.

### Collection, summary, and reporting of results

The results were summarized and reported descriptively, and are presented in Table 1. Implications for clinical practice and future research were also discussed.

**Table 1:** Summary of selected studies by inclusion and exclusion criteria

**Title**	**Cortical areas and polarities**	**Participants**	**Sessions (number and duration)**	**Combined intervention**	**Synopsis of results**	**Duration of the result**
Modulation of auditory temporal processing, speech in noise perception, auditory-verbal memory, and reading efficiency by anodal tDCS in children with Dyslexia^(19)^	Assembly (1): Anode-left temporoparietal region; and Cathode-right temporoparietal region.	17 children and adolescents	05 sessions, with a one-week interval between them	No intervention	- Significant effect on temporal resolution, speech perception, and auditory verbal memory tasks in the two active assemblies, speed and accuracy of texts, low-frequency words, and pseudowords compared to Sham and initial assessment.	- Effect observed immediately after stimulation.
	Assembly (2): Anode-left temporoparietal region;		(2 sessions in each assembly and 1 Sham session)	Combined	- Assembly (2) resulted in better temporal resolution, speech perception, and auditory memory outcomes than assembly (1).	
	and				- There was no difference in reading high-frequency words.	
	Cathode-right shoulder.					
Effects of a short and intensive transcranial direct current stimulation treatment in children and adolescents with developmental Dyslexia: A crossover clinical trial^(20)^	Anode-between the left occipitotemporal and left temporoparietal regions;	24 children and adolescents	05 consecutive sessions for 20 minutes.	- Word reading (high and low frequency), pseudowords, and text.	- Only in the active group: Positive effect on pseudoword reading speed, phonological working memory, and phoneme combination.	- Effect observed immediately after and one month after stimulation.
	and			- Cognitive-linguistic stimulation: operational phonological memory, combining phonemes, rapid automatic naming.	- No effect on reading speed of low and high-frequency words and reading accuracy of text, high and low-frequency words, and pseudowords.	
	Cathode-between the right occipitotemporal and right temporoparietal regions.					
Reading and phonological awareness improvement accomplished by transcranial direct current stimulation combined with phonological awareness training: A randomized controlled trial^(21)^	Anode-temporoparietal junction;	28 children and adolescents	15 sessions: 3 weekly sessions of 60 minutes each.	Cognitive-linguistic and reading training: “Gillon Phonological Awareness Training Programme”	- Positive effect on reading low- and high-frequency words and non-words for the Active and Sham groups from the 5^th^ session.	- Effect observed after the 5^th^, 10^th^, and 15^th^ sessions and 6 weeks later (follow-up).
	and				- Positive effect on reading non-words for Active from the 5^th^ and the 10^th^ for Sham.	
	Right cathode-temporoparietal junction.				- Active better at reading non-words from the 10^th^ session, immediately after, and at follow-up.	
					- Positive effect for Active in rhyme detection, from the 5^th^ session and at the end of the intervention and follow-up for Sham.	
	Sham				- Difference between groups: Active better at the end of the intervention, with maintenance at follow-up.	
					- Group effect for phoneme exclusion: Active better performance at the 5^th^ session; positive effect on Sham at the end and follow-up. No difference between groups.	
Beyond Reading Modulation: Temporo-Parietal tDCS Alters Visuo-Spatial Attention and Motion Perception in Dyslexia^(22)^	Assembly (1): Anode-left temporoparietal region;	10 children and adolescents	Two sessions, each lasting 20 minutes, and different setups, with a minimum 24-hour interval between them.	No intervention	- Positive effect on accuracy for the Group with assembly (1) in text reading.	- Effects seen immediately after stimulation.
	and			Combined	- Smaller positive effect and mean reaction time for the Group with assembly (1) on lexical decision tasks	
	Cathode-right contralateral region.				- No significant difference in reading speed and accuracy of low-frequency, high-frequency, and non-words.	
	Assembly (2): Anode-right temporoparietal region;				- No difference in phoneme matching tasks, working memory, and quick naming.	
	and				- Positive effects for assembly (1) with greater perception of movement and reduced visual-spatial attentional focus.	
	Cathode-left contralateral region.				- The main effect of stimulation on assembly (1) is more significant than in Group with assembly (2).	
Effects of a short, intensive, multi-session tDCS treatment in developmental dyslexia: Preliminary results of a sham-controlled randomized clinical trial^(23)^	Anode- parietal-occipital regions P7.	27 children and adolescents distributed in Active and Sham groups Cross-over.	05 consecutive sessions for 20 minutes.	No intervention	- Positive effect in the active group regarding the reading speed of non-words immediately after and 1 week after the end of treatment compared to baseline.	- Effects observed before, immediately after and 01 week after treatment.
				Combined		
	Cathode- – contralateral region (P8)					
	
	
Individual Differences Modulate the Effects of ETCC on Reading in Children and Adolescents with Dyslexia^(24)^	Anode-between the temporoparietal and left parietal regions;	26 children and adolescents distributed in Active and Sham groups	Eighteen sessions: 3 20-minute weekly sessions for 6 weeks and a minimum 48-hour interval between sessions.	Word reading training with acceleration and spelling.	- Positive effect regarding group, time, and word reading speed.	- Effects observed immediately after, one month after, and six months after.
	and				- Individual differences between participants of the Active group; less fluent and older subjects displayed a more significant change in word reading fluency at each time point than younger subjects.	
	Cathode-between the temporal and parietal regions on the right.					
	Sham					
Long-lasting improvement following ETCC treatment combined with training for reading in children and adolescents with Dyslexia^(25)^	Anode-between the temporoparietal and left parietal regions;	26 children and adolescents distributed in Active and Sham groups	Eighteen sessions: 3 20-minute weekly sessions for 6 weeks, with a minimum 48-hour interval between sessions.	Fast word reading (Tachistoscopic presentation)	- Active Group with a positive effect on the reading of low-frequency words one month after stimulation and pseudowords immediately one and six months after stimulation.	- Effects observed immediately after, one month after, and six months after.
	and			Cognitive training: phoneme-grapheme association^(26)^	- Active Group with better performance in reading low-frequency words and pseudowords.	
	Cathode-between the temporal and parietal regions on the right.				- Reading efficiency increased after 6 months in the stimulated group.	
	Sham					
Impact of Transcranial Direct Current Stimulation on Reading Skills of Children and Adolescents With Dyslexia^(27)^	Anode-left middle posterior temporoparietal region;	12 children and adolescents	5 consecutive days of stimulation of 30 minutes each session	No intervention	- Positive effect on pseudoword and text accuracy.	- Effect observed after the 5^th^ session.
	and			Combined	- There was no change in the number of correct letters, syllables, words, and reading time.	
	Cathode-right supraorbital region (FP2).					
Evidence for reading improvement following ETCC treatment in children and adolescents with Dyslexia^(26)^	Anode-between the temporoparietal and left parietal regions;	18 children and adolescents	18 sessions: 3 20-minute weekly sessions for 6 weeks	Fast word reading (Tachistoscopic presentation)	- Positive effect with decreased reading time of low-frequency words and pseudowords immediately and 1 month after the stimulation sessions.	- Effect observed immediately after and 1 month after the stimulation.
	and			Cognitive training: phoneme-grapheme association.	- Reduction of errors of immediate effect and 1 month after the stimulation.	
	Cathode-between the temporal and parietal regions on the right.					
	Sham					
Reading changes in children and adolescents with Dyslexia after transcranial direct current stimulation^(28)^	Assembly (1): Anode-left temporoparietal region;	19 children	One session with pre-evaluation, 20 minutes of stimulation, and post-evaluation carried out sequentially.	No intervention combined	- Positive effect with a difference between groups with assembly (1) in the improvement of text reading accuracy, phoneme combination time, and N-back verbal task.	- Effect observed 20 minutes after stimulation.
	and					
	Cathode-between the temporal and right parietal region.					

	Assembly (2) Anode-right temporoparietal region;					
	and					
	Cathode-left temporoparietal region.					
	Sham					
Improved reading measures in adults with Dyslexia following transcranial direct current stimulation treatment^(11)^	Anode-region of the left visual cortex (V5);	19 adults	5 sessions spread over 2 weeks (they did not mention defined intervals between them)	No intervention	- Positive effect with a difference between groups in reading speed and the RAN test of numbers and letter naming.	- Effects observed immediately after the 5^th^ session.
	and			combined		
	Cathode-region of the right orbitofrontal cortex.					
	Sham					

## RESULTS

### Population

Only one of the eleven analyzed studies investigated the stimulation’s effects in 19 adults^(11)^. In the remaining work, the studied population consisted of 207 children and adolescents, with ages ranging from 7.08 to 18 years.

### Electrode setup – brain location and currents’ polarity

The initial study, conducted in 2015^(11)^, positioned the anode electrode, responsible for promoting cortical excitability, in the left visual cortex region (V5), while the cathode, responsible for reducing cortical excitability, was positioned in the right orbitofrontal cortex region and compared with a sham group.

Three studies replicated this setup and compared active and sham groups^(24-26)^, the anode electrode was placed in the left temporoparietal and parietal region, and the cathode in the right temporal and parietal region.

Four studies positioned the electrode in the left temporoparietal region, with the cathode in the right temporoparietal region (contralateral region)^(19,21,22,28)^. One of these studies implemented the setup described previously and compared it with a placebo group^(21)^; Study 1 compared this setup with another group in which the cathode electrode was placed in the right shoulder region; another study^(22)^ compared the effects of polarity in two groups, one with the setup with the anode in the left region and cathode in the homologous right region, and another group with the inverse setup (anode in the right region); yet another study^(28)^ also performed the comparison to observe the effects of polarities and hemispheres, but also added a third placebo group.

A cross-over methodology study positioned the anode electrode between the left occipitotemporal and left temporoparietal regions, with the cathode in the homologous contralateral region, comparing the active and sham conditions^(20)^.

Another study using the cross-over methodology examined the effects of setting up the anode electrode in the left occipitoparietal region and the cathode in the contralateral region, comparing the active and sham groups^(23)^.

### Number of sessions

The studies varied in the number of sessions performed: two had a single session^(22,28)^, while three had five consecutive sessions^(20,23,27)^. One specific study^(11)^ had five sessions spread over two weeks without specifying the intervals. Furthermore, one study had five sessions, with one-week intervals between them^(19)^; one had 15 sessions distributed over three weekly sessions^(21)^, and yet another had 18 sessions, three weekly sessions for six weeks, with a minimum interval of 48 hours between them^(24-26)^.

## THERAPY COMBINED WITH TDCS

Most studies did not combine Transcranial direct current stimulation with other therapies. Only five clinical trials did so, using stimulation and reading and cognition training^(20,21,24-26)^.

### Reading rate and accuracy outcomes

Detailed outcome results can be viewed in the results table. However, it is crucial to interpret them cautiously due to methodological variations in clinical trials, including electrode setup, number of sessions, and whether or not combined therapy is used. In general, the groups subjected to active and anodal stimulation on the left showed better results in reading rate and accuracy tasks. We summarize the outcomes below:

- Improved text reading speed: observed in 2 studies^(11,19)^;
- Improved text reading accuracy: observed in 5 studies^(11,19,22,27,28)^;
- Improved word reading speed: observed in 3 studies^(19,24,26)^;
- Improved word reading accuracy: observed in 6 studies^(19,21,24-27)^;
- Improved pseudoword reading speed: observed in 4 studies^(19,20,23)^;
- Improved pseudoword reading accuracy: observed in 5 studies^(19,21,25-27)^.

### Other outcomes

Besides reading outcomes, improvements were observed in other aspects:

- Temporal resolution, speech perception, and auditory memory^(19)^;
- Working memory and phoneme combination task^(20)^;
- Phonological awareness tasks^(21)^;
- Lexical decision, movement perception, and attentional focus^(22)^;
- Time spent performing phoneme combination tasks and improved skills in the n-back verbal task^(28)^;
- Improvement in quickly naming numbers and letters^(11)^.

## DISCUSSION

Historically, cognitive-linguistic and reading skills were stimulated in Developmental Dyslexia (DD) cases^(6,7)^, mainly to adapt decoding and reading fluency to facilitate academic learning. However, in DD, the typical persistence of the phonological processing deficit tends to delay achieving positive results from clinical interventions^(1,7)^. Furthermore, the effects of intervention programs reported in research have been minor^(7,29)^.

From 2015 onwards, transcranial direct current stimulation (tDCS) was adopted in clinical trials, with the first study that looked at the effect of current on reading in dyslexic adults^(11)^. Other works followed this study, with different age groups and number of participants, assembly of electrodes and application areas, combination of other therapies on application, number of sessions, and, consequently, results observed. Although they varied in all these aspects, the clinical trials with tDCS analyzed in this review reported positive effects on the speed or accuracy of reading the linguistic items presented in the immediate^(11,19-23,26-28)^ or longitudinal assessments^(24,25)^.

The effects reported as a result of the application of tDCS combined or not with other therapies have positively increased reading speed and accuracy and shortened the stimulation period, which ranged from two^(19,22)^ to 18 sessions^(24-26)^, in a maximum time of one and a half months, in order to observe positive outcomes.

This analysis also showed that they produced different outcomes even when they were identical regarding combined interventions and participant age^(24-26)^. This result suggests that individual differences in patients must be considered, as was later demonstrated by a study^(24)^, who retroactively verified which participants benefited the most from the therapy applied: the older people, with a higher IQ, and with low reading rate and accuracy values at the beginning of the intervention.

Considering that individual differences may have influenced the reported results, we can remember that DD theories suggest different types of dyslexia according to their manifestations and hypotheses of deficits in different cortical and subcortical areas. The literature reports^(30)^ three main DD types, per deficit reading route: phonological dyslexia, in which we observe deficits mainly related to phonological processing, with significant difficulties in decoding new and infrequent words; visual dyslexia, in which visual processing is altered, with difficulty in automatic word recognition, slow decoding, even in high-frequency words or with possible multiple representations; the mixed type, in which phonological and lexical reading routes would be impaired. Both routes must be used efficiently for good reading^(31,32)^. None of the studies indicated that they had considered reading routes when setting up their protocols, which may partly explain the significant variability in the results reported.

The choice of the location to receive tDCS is based on studies that highlight enhanced activation of brain areas, particularly posterior areas of the left hemisphere, in individuals with typical reading performance^(33)^. Thus, the modulation of neuronal excitability through tDCS, promoting greater activation of these areas, can induce changes in brain plasticity^(34)^ and modify behavioral, cognitive, and perceptual functions^(35,36)^.

In DD protocols, studies have shown that increasing cortical excitability by applying the anode electrode to the left in areas involved in reading circuits (temporoparietal, occipitotemporal, and occipitoparietal areas) promoted positive effects on reading^(11,19-28)^. Brain plasticity, the ability of the brain to adapt and change over time in response to external or internal stimuli, was evidenced by the results of these studies.

These studies highlighted the stimulation of posterior areas of the left hemisphere. They suggested that tDCS can be an effective complementary strategy to facilitate the activation of reading-related brain areas, enhancing reading development. Some of these studies have also reported the combination of tDCS with therapies from different stimulation approaches to improve reading^(20,21,25,26,28)^.

The role of the cathode electrode in improving reading is still under discussion. At the same time, most studies placed the cathode electrode in the contralateral homologous region to promote greater lateralization of the function. One study in which it was placed in the contralateral shoulder did not observe significant difference in reading but brought improvements in other parameters such as temporal resolution, speech perception and auditory memory^(19)^. The analysis of the studies consulted indicated that the neuromodulation technique alone improved the performance of individuals with DD since six of the studies did not combine tDCS with other interventions. However, all indicated some improved reading parameters studied.

When we analyzed the results of studies that combined tDCS with the stimulation of reading or cognitive-linguistic skills, we found better performance in the group that received therapy plus active tDCS, showing that this technique was a complementary tool that enhanced reading compared to sham. We did not identify studies comparing tDCS alone with the combined use of tDCS with other stimulations, which limits this discussion.

Finally, we should also think about the long-term effects produced by tDCS, especially when it comes to developing individuals, such as children and adolescents, most of whom participate in the studies mentioned here. To this end, it is essential to understand the metaplastic effects of tDCS and its ability to influence the brain’s response to future stimuli^(37)^. The tDCS may precondition the brain to be more receptive to later stimuli, making it more susceptible to long-lasting changes in neuronal plasticity^(38)^. The study by Constanzo et al.^(25)^ was the first to demonstrate long-term effects, probably due to the metaplasticity caused by tDCS, after observing significant improvements in reassessment after six months, also observed later^(12,24)^.

The analysis of the results of this review did not aim to quantify the effects achieved due to the different parameters used in the research. However, it pointed out the positive effects of tDCS on reading skills in DD. Considering the slow DD clinical development^(1)^, the post-stimulation positive effects, combined or not, are promising in terms of results and durability. New experiments should investigate parameters such as electrode application site, number of sessions, combination of other therapies, and DD diagnostic criteria, as the varying results identified show that all these parameters are essential for studying the application of tDCS in the rehabilitation of DD.

### Study limitations

Most studies have been conducted with small samples, which restricts the generalizability of results. Furthermore, the different methods described hinder the performance of a meta-analysis.

## CONCLUSION

The findings of the present systematic review showed that tDCS, isolated or combined with interventions of cognitive-linguistic stimulation of phonological processing and reading training, promoted positive effects, in the short, medium, and long term, on the speed and accuracy of reading and related skills (for example, phonological awareness and visual and speech perception).

This study underscores the need for further research to allow the observation and measurement of effects, depending or not on the location of each electrode, the frequency of stimulation sessions, and the choice of the best combined intervention program in stimulations with tDCS in DD. Future studies should consider the protocols used in diagnosing DD, the specific types of DD, and the most impaired reading routes to develop and use more accurate stimulation protocols.
